# Role of frailty assessment in selection for cancer surgery

**DOI:** 10.1093/bjsopen/zrac117

**Published:** 2022-10-18

**Authors:** William H Allum

**Affiliations:** Department of Academic Surgery, Royal Marsden NHS Foundation Trust, London, UK

The effect of the ageing population on cancer management is well established with its significant effect on all aspects of medical care. In their study, Layfield *et al.*^1^ reviewed their experience of changes in patient treatment determined by the multidisciplinary team in colorectal cancer over a 14-year interval. There were several changes during this time, including colonoscopic screening for colorectal cancer, and the effect of such changes is borne out in this study. It is apparent that with the evolution of multidisciplinary working, the approach to patients presenting with colorectal cancer has changed, with appropriate assessment and recommendation for different therapeutic options. Although, as the authors point out, surgery has been the mainstay, changes in better understanding of colorectal cancer biology have tailored oncological treatments. The study clearly shows how changes in different interventions have resulted in changes in outcome, which has occurred across all ages.

A focus in this study is the effect of the initiatives when assessed by age. In older patients with cancer there is often a close interrelationship between effect of the cancer and co-morbidity, which may be pre-existing, impacted by the cancer itself, or related to the cancer treatment options. In this study the assessment for fitness for treatment has been based upon reasons for not operating, which included a risk assessment scoring system as well as cardiopulmonary exercise testing. These assessments reflect existing co-morbidity, which can be improved with pre-habilitation support, including optimization of medication, stopping smoking, and encouraging exercise^2^. There are limitations with these assessments, but they have a role in providing information on expected recovery after major surgery.

Latterly, greater understanding of performance status and cognitive function has been shown to independently influence treatment selection and outcome^3^. In this study the authors acknowledge that for frail patients best management can be more difficult to determine. The use of frailty scores, which can be simply completed during a clinical consultation, add additional information to inform treatment decisions. Such scores include assessments of ability to complete daily activities, mobility, dependency, and cognitive function. In a study conducted at the Royal Marsden NHS Foundation Trust in London, where therapists, including dieticians, physiotherapists, and occupational therapists, together with anaesthetists provided a frailty package implemented before admission for cancer surgery, length of stay reduced from 13 to 8 days and the postoperative morbidity score was reduced from 46 per cent to 17 per cent (W. H. Allum, in preparation). In a small randomized clinical trial, a similar approach significantly reduced length of stay from 8.2 to 5.9 days and this included careful management with care of the elderly specialists^4^.

This study shows how appropriate personalized treatment has an overall improvement in 90-day survival after surgery as well as an increase in median survival for the elderly cohort. This is reflected in the increase in the number of patients who did not undergo surgery. It would be interesting to know of any changes in patient-reported outcomes for this group. The authors consider that it may be possible to increase the patients offered resection in the context of improved perioperative care. The inclusion of frailty assessment should be considered part of this package of care alongside co-morbidity assessment to advise likely recovery and convalescence to enhance individual decisions by patients and their families on treatment options.
